# Prevalence of APOL1 Risk Variants in Afro-Descendant Patients with Chronic Kidney Disease in a Latin American Country

**DOI:** 10.1155/2019/7076326

**Published:** 2019-12-18

**Authors:** Carlos E. Duran, Alejandro Ramírez, Juan G. Posada, Johanna Schweineberg, Liliana Mesa, Harry Pachajoa, Mayra Estacio, Eliana Manzi, Vanessa Aros, Lorena Díaz, Victor H. Garcia

**Affiliations:** ^1^Department of Nephrology, Fundacion Valle del Lili, Cali, Colombia; ^2^Health Sciences Faculty, Icesi University, Cali, Colombia; ^3^Department of Basic Medical Sciences, Center for Research on Congenital Anomalies and Rare Diseases (CIACER), Icesi University, Cali, Colombia; ^4^Clinical Research Center, Fundación Valle del Lili, Cali, Colombia

## Abstract

**Introduction:**

In Colombia, the genetic background of the populations was shaped by different levels of admixture between Natives, European, and Africans. Approximately 35.363 patients have diagnosed chronic kidney disease and according to population studies, 10.4% of these patients are Afro-descendant. We aim to assess the frequency of APOL1 variants G1 and G2 in Afro-descendant patients with ESRD treated at la Fundacion Valle del Lili University Hospital in Cali, Colombia.

**Methods:**

This is an observational cross-sectional study. Afro-descendant patients with ESRD in waitlist or recipients of kidney transplant were evaluated. Clinical data were collected from the electronic medical records. Genotyping was carried out by amplification of the exon 7 of the APOL1 gene. For the identification of risk genotypes, the bioinformatics tool BLAST was used.

**Results:**

We enrolled 102 participants. The frequency of APOL1 risk variants was 67.2%, in which 24.5% (*n* = 25) were G1 heterozygous and 5.8% (*n* = 6) were G2 heterozygous and 37% of the patients had high-risk status with two alleles in homozygous (G1/G1 = 21 and G2/G2 = 3) or compound heterozygote (G1/G2 = 14) form.

## 1. Introduction

The APOL1 gene is located at chromosome 22 and encodes the homonymous protein apolipoprotein L1, which is expressed in multiple tissues and cell types. It circulates coupled to high-density lipoproteins (HDL). Two common allelic variants have been recognized for this gene: G1 and G2, which have been stated to be protective factors against *Trypanosoma brucei rhodesiense* infection [1].

In 2010, Genovese et al. described that the focal segmental glomerulosclerosis (FSGS) and hypertension-attributed end-stage renal disease (ESRD) are associated with two independent sequence variants in the APOL1 [2] among African American individuals. Furthermore, the association with other nondiabetic renal diseases has also been described. Examples include the human immunodeficiency virus- (HIV-) associated nephropathy, lupus nephritis, sickle cell nephropathy, focal global glomerulosclerosis, and rapid failure of transplanted kidneys from donors with APOL1 high-risk variants [3].

The mechanism by which APOL1 causes kidney disease is not fully understood. Previous studies have suggested that APOL1 risk variants cause podocyte injury partly through enhancing endoplasmic reticulum stress [4]. It has also been described that the high-risk variants are associated with impaired mitochondrial function with reduced maximal respiration rate, respiratory capacity, and membrane potential. Other authors have shown that APOL1 has a splice variant (termed B3), and the risk-variant G2 activates the NLRP3 inflammasome. Nevertheless, the decline in renal function attributed to *APOL1* risk variants is dependent upon plasma levels of the soluble urokinase plasminogen activator receptor (suPAR) because APOL1 G1 or G2 augments *α*v*β*3 integrin activation and causes proteinuria in mice in a suPAR-dependent manner [5]. APOL1 also interacts with VAMP8-coated vesicles, inducing a reduction in local dynamics of the C-terminal which in turn causes progressive and repeated injury, leading to chronic kidney disease in susceptible African Americans subjects [6].

Recently, two new mechanisms that may serve as the foundation for new and promising treatments have been described. The APOL1 risk alleles activate the protein kinase R (PKR) which in turn induces glomerular injury and proteinuria [7]. Moreover, the APOL1 may function similarly to the LD-associated protein CIDEA, which has an amphipathic helix that facilitates embedding in the phospholipid monolayer and binding to phosphatidic acid, and altered association of APOL1 with LDs may alter APOL1 delivery to mitochondria, possibly controlling cytotoxicity [8].

The prevalence of mutations of the APOL1 gene among Afro-descendant patients with chronic kidney disease for the G1 and the G2 variants can be of 20–22% and 13–15%, respectively [9].

The genepool of Colombian population results from the admixture of Native Americans, Europeans, and Africans [10]. It is currently estimated that 10.4% of the Colombian population is Afro-descendant [11]. We aim to assess the frequency of APOL1 variants G1 and G2 in Afro-descendant patients with ESRD treated at la Fundacion Valle del Lili University Hospital in Cali, Colombia.

## 2. Materials and Methods

We performed an observational, cross-sectional study of Afro-descendant adult patients with end-stage renal disease who were on the waitlist or were already recipients of a kidney transplant. Patients with acute kidney failure, combined kidney transplantation, cognitive impairment, or psychiatric disease that would affect their ability to voluntarily consent to participate in the study were excluded. The race was assessed by self-recognition of the race and physician-assessed classification of skin color.

Blood samples were obtained from participants after signing the informed consent. Data were collected by interview and review of medical records from patients attended at our institution from January to November 2017. The institutional review board approved the study protocol.

### 2.1. Molecular Analysis Methodology

Genomic DNA extraction was performed using E.Z.N.A® Tissue DNA Kit (Omega Bio-Tek) according to manufacturer's instructions. Quantity and purity of gDNA extracted were assessed using a Nanodrop2000® UV-Vis spectrophotometer (Thermo Fisher Scientific). Amplification of gDNA was performed by PCR. The set of primers (amplifying a 421 bp in the exon 7 of the *APOL1* gene), amplification reaction setup, and thermocycling conditions have been described in a previous study [13]. PCR products were run on an agarose gel to verify the size of the amplified fragment: 5 *μ*l of each PCR reaction was analyzed in a 1% agarose gel containing 0.5% ethidium bromide and was visualized by UV illumination. The PCR products were purified using an E.Z.N.A. Cycle-Pure Kit (Omega Bio-Tek) and sequenced using BigDye chemistry in an ABI 3500 automated system (Applied Biosystems) according to manufacturer's instructions.

### 2.2. Statistical Analysis

The categorical variables were summarised in proportions, and the continuous variables were expressed as the mean and standard deviation (SD). The normality of the age and age of dialysis initiation was evaluated by the Shapiro–Wilk test. Data were analyzed using STATA. APOL1 variants were classified as G1 heterozygous, G1 homozygous, G2 heterozygous, G2 homozygous, and mixed G1/G2 variants. *APOL1* high-risk status was defined as the presence of 2 risk alleles (G1/G1, G2/G2, or G1/G2) versus the low-risk status, defined as having 1 or 0 risk variants (G1/G0, G2/G0, or G0/G0).

## 3. Results

We enrolled a total of 102 patients, of which 56% (*n* = 57) were female. The mean age was 48 years (SD 13 years). There were 44 patients in the transplant waitlist and 58 in the posttransplant period. The mean time of dialysis before kidney transplant was 4 years.

The demographic characteristics of the group are described in Table 1. The etiological cause of ESRD was as follows: unknown in 82%, diabetic nephropathy in 5%, lupus nephritis in 5%, polycystic disease in 4%, and others in 4%. The comorbid condition more frequently found was arterial hypertension in 88% of patients.

We found that 37% of patients had *APOL1* high-risk status in which two alleles were in homozygous (G1/G1 = 21 and G2/G2 = 3) or compound heterozygote (G1/G2 = 14) form and 63% had *APOL1* low-risk status. There were no significant differences in the age of dialysis initiation, comorbidities, or the etiology of chronic kidney disease between the two groups.

The frequency of the APOL1 G1 and APOL1 G2 risk allele variants is described in Table 2. The APOL1 risk variants were found in 69 patients (67.2%). Of these, 24.5% (*n* = 25) were G1 heterozygous and 5.8% (*n* = 6) were G2 heterozygous.

The geographical distribution of our findings is described in Table 3. The highest frequency of high-risk variants (G1/G1, G2/G2, or G1/G2) was found in the population of the South West. The frequency of G1 risk genotype was the same in the South West and West regions. Patients homozygous for G2 were located exclusively in the South West region.

## 4. Discussion

This study shows that 37% of the Afro-descendant patients with chronic kidney disease had an APOL1 high-risk allele. Our prevalence rate is similar to that reported in other studies involving ESRD patients. Reports from North America have shown the frequency of APOL1 to be of 20%–39% [13, 14]. In contrast, reports from Asiatic and some Latin American countries have shown much lower frequencies (1.9–9.4%) [15, 16].

Previous studies have shown that the G1 and G2 APOL1 risk alleles were most common in Western and Southern Africa [2, 17]. The analysis of HapMap populations showed that the G1 and G2 variants were present in approximately 38% and 8% of the Yoruba population from Nigeria in West Africa. The PAGE study included 99 unique populations (51,698 participants), and the highest frequencies of the APOL1 haplotype were reported in African American, sub-Saharan African, and Western African populations (11 to 32%) [18].

Thomson et al. reported that high frequencies of G1 were concentrated in West Africa, whereas G2 had a more uniform distribution between continents, worldwide [19]. The West African distribution of G1 may reflect the distribution of *Trypanosoma brucei gambiense* type 2, while the G2 variant is more potent against *Trypanosoma rhodesiense* [9]. Our study found a higher frequency of G1 as in West Africa.

The African ancestry in Colombia comes from Western Africa and West-Central Africa [10, 20, 21]. In the sixteenth century, the trans-Atlantic slave trade brought millions of Africans to the New World. The slaves that came to the Caribbean and western Pacific coasts were originated from Upper Guinea, Cabo Verde, Lower Guinea, and Angola. Therefore, the genetic background of the Colombian population was shaped by different levels of admixture between Natives, Europeans, and Africans. African ancestry is more common in the Pacific and west subregion populations [10].

Our study is the first to explore APOL1 risk allele status among Colombian patients with known ESRD. In 2018, there were 35,363 patients diagnosed with chronic kidney disease (CKD) throughout the country [22]. Although we included only patients from the southwestern region of the country, we believe that the impact of the APOL1 mutation could be higher in the whole population, as the last census reported that there is an estimate of 4.311.757 Afro-Colombian inhabitants in the country corresponding to 10.4% of the population [23].

APOL1 risk alleles are associated with higher systolic blood pressure and earlier hypertension diagnoses in young Afro-Americans [24]. In our study, 88% of our patients had a diagnostic of arterial hypertension. The association between mutations in APOL1 and hypertension is complex, and it could be explained by three possible relationships: first, APOL1 variants lead to CKD which in turn leads to hypertension. Second, APOL1 variants contribute to hypertension and to a second hit which leads to CKD. Third, APOL1 variants lead to both hypertension and CKD and the latter is impacted both by hypertension and the second hit [25]. At the moment, more research is needed to clarify the complex relationships between APOL1, renal function, and blood pressure.

Risk alleles of the APOL1 gene have been associated with higher risk-adjusted odds of earlier initiation of chronic hemodialysis; however, in our study, we did not find statistically significant differences in the age of onset of dialysis. Our results differ from those reported previously, and we believe that this is in part because only nondiabetic African American patients were included in those groups [13, 14].

Although 56.8% of our patients were in the posttransplant period, we did not perform analyses regarding allograft survival times. Previous reports have found a relationship between the presence of APOL1 mutation and outcomes in kidney transplantation. For example, Freedman et al. found that patients with the two high-risk alleles of the APOL1 gene had shorter allograft survival [26]. These kinds of analyses are being performed at our lab, and results will be published in a later time.

Our study is not without limitations, and results should be interpreted in the context of the study design. First, the race was not assessed by mitochondrial haplogroup or autosomal genetic ancestry. Second, the size of the cohort was determined based on the available budget for the molecular analysis, which limits the applicability of our results. Despite our limitations, we believe that our analysis advances the knowledge of the APOL1 mutations and its impact on outcomes among the Afro-descendant population. However, a furthermore extensive multicenter study should be performed to better understand the frequency and role of these mutations among ESRD patients in Colombia.

## Figures and Tables

**Table 1:** Baseline characteristics.

Characteristics	All patients *n* = 102	Low risk *n* = 64	High risk *n* = 38	*P* value
Age, year, mean (SD)	48 (13)	49 (13)	46 (12)	0.2859
Range	19–77	20–77	19–75	
Age at start of dialysis, mean (SD)	40 (13)	41 (14)	38 (12)	0.4210
Range	6.3–69	6.3–69	13.3–65	
Sex, male, *n* (%)	58 (57)	35 (55)	23 (60)	0.565
Comorbid condition, *n* (%)				
Arterial hypertension	90 (88)	56 (87)	34 (89)	0.765
Diabetes	7 (6.2)	5 (8)	2 (5)	0.622
Peripheral vascular disease	16 (16)	13 (21)	3 (8)	0.083
Autoimmune disease	6 (5.8)	6 (9.7)	0	0.050
Etiology ESRD, *n* (%)				0.108
Unknown	84 (82)	48 (75)	36 (94)	
Lupus nephritis	5 (5)	5 (8)	0	
Diabetic nephropathy	5 (5)	5 (8)	0	
Polycystic disease	4 (4)	3 (4.7)	1 (2.6)	
Others	4 (4)	3 (4.7)	1 (2.6)	

**Table 2:** Allele and genotype frequencies.

Allele G1 *rs73885319*	Allele G2 *rs71785313*	Genotype	All patients (*n* = 102) *n* (%)
Heterozygous	—	G1	25 (24.5)
Homozygous	—	G1	21 (20.5)
Heterozygous	Heterozygous	G1/G2	14 (13.7)
—	Heterozygous	G2	6 (5.9)
—	Homozygous	G2	3 (2.9)

**Table 3:** Geographic distribution of allele and genotype frequencies.

Origin	G1/G0	G2/G0	G1/G1	G2/G2	G1/G2	All patients (*n* = 102) (%)
South West, *n*	11	0	9	3	10	33 (32.3)
Pacific region, *n*	1	—	3	—	—	4 (3.9)
West, *n*	13	6	9	—	4	32 (31.4)

## Data Availability

The data used to support the findings of this study are included within the article.
