# Corrigendum to “Prevalence of APOL1 Risk Variants in Afro-Descendant Patients with Chronic Kidney Disease in a Latin American Country”

**DOI:** 10.1155/2020/8706297

**Published:** 2020-11-17

**Authors:** Carlos E. Duran, Alejandro Ramírez, Juan G. Posada, Johanna Schweineberg, Liliana Mesa, Harry Pachajoa, Mayra Estacio, Eliana Manzi, Vanessa Aros, Lorena Díaz, Victor H. Garcia

**Affiliations:** ^1^Department of Nephrology, Fundacion Valle del Lili, Cali, Colombia; ^2^Health Sciences Faculty, Icesi University, Cali, Colombia; ^3^Department of Basic Medical Sciences, Center for Research on Congenital Anomalies and Rare Diseases (CIACER), Universidad Icesi, Cali, Colombia; ^4^Clinical Research Center, Fundación Valle del Lili, Cali, Colombia

In the article titled “Prevalence of APOL1 Risk Variants in Afro-Descendant Patients with Chronic Kidney Disease in a Latin American Country” [1], the authors Harry Pachajoa and Lorena Diaz were affiliated to Department of Basic Medical Sciences, Center for Research on Congenital Anomalies and Rare Diseases (CIACER), Icesi University, Cali, Colombia, which is incorrect. The correct affiliation for the authors Harry Pachajoa and Lorena Diaz is Department of Basic Medical Sciences, Center for Research on Congenital Anomalies and Rare Diseases (CIACER), Universidad Icesi, Cali, Colombia, which is listed above.
